# Racially discriminatory experiences among older chronically ill people of Turkish origin in Germany

**DOI:** 10.1093/eurpub/ckac129.730

**Published:** 2022-10-25

**Authors:** H Tezcan-Güntekin

**Affiliations:** 1 Alice Salomon University of Applied Science, Department of Health and Education, Berlin, Germany; 2 Charité Berlin, Berlin School of Public Health, Berlin, Germany

## Abstract

**Background:**

Racism in health care is rarely clearly identified as such in Germany. Barriers to access are more often addressed, but people's experiences of discrimination rarely receive attention (Schellenberg & Tusch 2021, Kristiansen 2016). Internationally, racism is recognized as a determinant of health and there is a discourse that it must be addressed in order to achieve health equity (Weil 2022). This qualitative secondary data analysis focuses on racially discriminatory experiences among older chronically ill people of Turkish origin in the context of medical and drug care in Germany.

**Methods:**

11 expert interviews and 11 problem-centred interviews with chronically ill people of Turkish origin and their relatives in Germany were conducted in the MedikaMig-Project with regard to care practices, continuity of drug care and polypharmacy and analysed with structuring qualitative content analysis (Mayring 2015).

**Results:**

The analysis yielded 7 superordinate categories and 27 subcategories, identifying racial discrimination in access to care, the treatment situation and communication, and in the consideration of transnational lifestyles. Patients are often helpless, sometimes trying to put their experiences into perspective or to be treated by Turkish doctors in order to avoid these discriminatory experiences.

**Conclusions:**

Racial discrimination is pervasive in health care and should not be hidden behind other terms that mask discrimination. An intersectional approach allows us to understand which individuals are particularly affected and what they need to be protected from racism in health care.

**Key messages:**

• Structural racism in health care needs to be clearly named and examined from an intersectional perspective in further research projects.

• Elderly patients with Turkish origin need empowerment and contact persons after racist experiences to find ways to deal with discriminating experiences that are sometimes perceived as traumatic.

